# Fluorescence-tagged metallothionein with CdTe quantum dots analyzed by the chip-CE technique

**DOI:** 10.1007/s11051-015-3226-8

**Published:** 2015-10-28

**Authors:** Ewelina Guszpit, Sona Krizkova, Marta Kepinska, Miguel Angel Merlos Rodrigo, Halina Milnerowicz, Pavel Kopel, Rene Kizek

**Affiliations:** Department of Biomedical and Environmental Analysis, Faculty of Pharmacy, Wroclaw Medical University, Borowska 211, 50-556 Wroclaw, Poland; Department of Chemistry and Biochemistry, Faculty of Agronomy, Mendel University in Brno, Zemedelska 1/1665, 613 00 Brno, Czech Republic; Central European Institute of Technology, Brno University of Technology, Technicka 3058/10, 616 00 Brno, Czech Republic

**Keywords:** Bioconjugation, Chip-CE, Metallothionein, Quantum dots, Biomarkers, Health effects

## Abstract

**Abstract:**

Quantum dots (QDs) are fluorescence nanoparticles (NPs) with unique optic properties which allow their use as probes in chemical, biological, immunological, and molecular imaging. QDs linked with target ligands such as peptides or small molecules can be used as tumor biomarkers. These particles are a promising tool for selective, fast, and sensitive tagging and imaging in medicine. In this study, an attempt was made to use QDs as a marker for human metallothionein (MT) isoforms 1 and 2. Four kinds of CdTe QDs of different sizes bioconjugated with MT were analyzed using the chip-CE technique. Based on the results, it can be concluded that MT is willing to interact with QDs, and the chip-CE technique enables the observation of their complexes. It was also observed that changes ranging roughly 6–7 kDa, a value corresponding to the MT monomer, depend on the hydrodynamic diameters of QDs; also, the MT sample without cadmium interacted stronger with QDs than MT saturated with cadmium. Results show that MT is willing to interact with smaller QDs (blue CdTe) rather than larger ones QDs (red CdTe). To our knowledge, chip-CE has not previously been applied in the study of CdTe QDs interaction with MT.

**Graphical Abstract:**

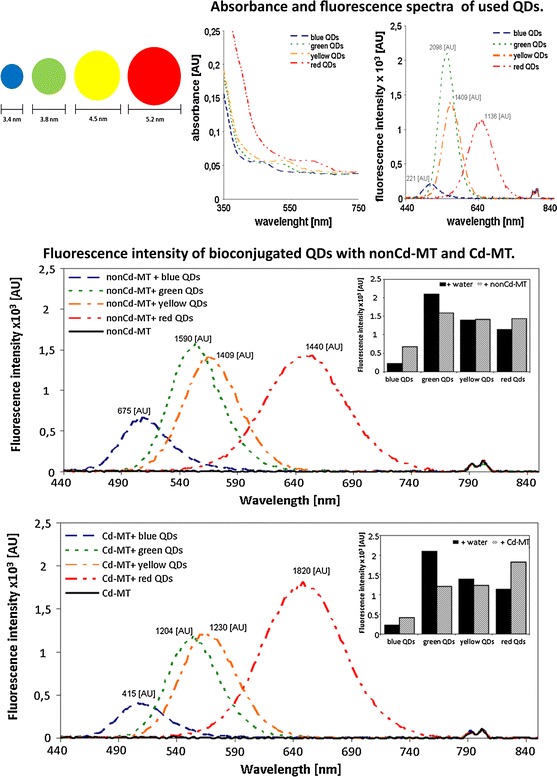

**Electronic supplementary material:**

The online version of this article (doi:10.1007/s11051-015-3226-8) contains supplementary material, which is available to authorized users.

## Introduction

### Metallothionein: structure and properties

MT was first isolated from a horse kidney in 1957 by Margoshes and Vallee as a cadmium binding protein of low molecular weight (about 6 kDa) (Margoshes and Vallee 1957). Nearly 30 % of the protein consist of cysteines, each containing one SH group which gives them the ability to bind metal ions (Bremner and Beattie 1990; Duncan and Stillman 2006). MT plays an important role in the homeostasis of heavy metals (Coyle et al. 2002; Sabolic et al. 2010), protection against oxidative stress (Bizon et al. 2011; Ruttkay-Nedecky et al. 2013), and DNA damage (Cai et al. 2000; Chubatsu and Meneghini 1993). MT is involved in the regulation of gene expression and transcription (Andrews 2000; Davis and Cousins 2000), enzyme activation (Krizkova et al. 2009a; Zalewska et al. 2014), and other biological processes.

Four major isoforms have been identified in mammals: MT-1, MT-2, MT-3, and MT-4 (Simpkins 2000). The best known isoforms MT-1 and MT-2 are expressed in nearly all types of tissues. Thanks to the presence of sulfur-based metal clusters in the particle, MT can bind with 7 Zn (II), Cd (II) or 12 Ag (I), Cu (I) ions per molecule. The MT binding affinity to metal ions differs between various metals or is metal dependent. For rat liver MT, the relative affinities of MT for Cd (II) were higher than for Zn (II) (Waalkes et al. 1984), while higher affinities of MT than for Cd (II) were found for Cu (I), Hg(II) and Ag (I) (Nielson et al. 1985; Hamer 1986). This indicates that Cd (II) ions can displace Zn (II) from MT structure. The main roles of MT-1 and MT-2 are detoxification of heavy metals from the body and protection against oxidative compounds (Bizon et al. 2011; Kagi and Schaffer 1988; Krizkova et al. 2010a; Moffatt and Denizeau 1997). MT-3 has been found mainly in the central nervous system (brain tissue), but also in kidneys, the heart, and reproductive organs, whereas MT-4 occurs in epithelial cells (Ruttkay-Nedecky et al. 2013; Vasak and Meloni 2011; Wang et al. 2006). MT-3 was first identified by Uchida et al. and originally termed a growth inhibitory factor (Uchida et al. 1991). MT-3 plays an important role not only in brain diseases, particularly in Alzheimer’s disease, but also in Parkinson’s disease and cognitive disorders (Carpene et al. 2007; Craig-Schapiro et al. 2009; Manso et al. 2012; Vasak and Meloni 2011).

Changes in the expression of MT could be a prognostic marker in the development of tumor malignancies, such as breast, prostate, ovary, head, and neck, or small-cell lung cancer, melanoma, and soft tissue sarcomas (Cherian et al. 2003; Krizkova et al. 2009b; Krizkova et al. 2010b; Krizkova et al. 2011; Namdarghanbari et al. 2011). However, the expression level of this protein is not universal for all human tumors and may depend on the state of tumor proliferation. Over the last decade, some data have appeared illustrating that induction of MT can be used as an adjunct in cancer chemotherapy, to prevent the toxicity caused by gamma radiation or the action of cisplatin, as well as by other chemotherapeutic agents (Doz et al. 1993; Karotki and Vasak 2009; Koberle et al. 2010; Sogawa et al. 2013). Previously published papers show that there are significant problems with the identification and monitoring of MT expression and its concentration. The challenge is to develop techniques for a selective, sensitive, and rapid labeling of this protein.

### Preparation of QDs and their application

Because of the changes in structural and functional properties, resulting from reductions in their size, nanomaterials have become very popular. An additional advantage of NPs is the ability to obtain biocompatible molecules in nanoscale which allows for quick and easy use of NPs in the field of science and industry (Niemeyer 2001; Rana et al. 2010; Roco 2003; Salata 2004; Sanvicens and Marco 2008). Of equally large significance is the development of fluorescent labeling techniques, due to the possibility for their use in treatment of individual marking (targeted therapy) (Brannon-Peppas and Blanchette 2012; Liu et al. 2007), molecular imaging, and also in chemical and biochemical assays (Drbohlavova et al. 2009; Rao et al. 2007; Terai and Nagano 2013). With the evolution of nanotechnology, the search for new biocompatible markers has led to the emergence of luminescent materials with properties known as semiconductor quantum dots (QDs) and lanthanide-doped nanocrystal (Drbohlavova et al. 2009; Gao et al. 2005; Smith et al. 2004; Wang and Li 2007).

QDs are nanocrystals with structures containing metal atoms of groups II-VI (e.g., CdS, CdTe, ZnSe) or III-V (e.g., InP, InAs) of the periodic table (Chan et al. 2002). Luminescence of the particles is due to quantum effects associated with changes permitted in electron states. QDs are characterized by the emission of visible light (depending on the size of the NPs) after ultraviolet irradiation (UV) (Drbohlavova et al. 2009; Michalet et al. 2005; Smith et al. 2004).

During the process of QDs preparation, it is possible to control such conditions as time, temperature, and type of ligand, thus obtaining the desired crystallite size and shape. The spectroscopic properties of QDs strongly depend on the size and shape of the crystallites (Alivisatos 1996; Drbohlavova et al. 2009; Gao et al. 2005). Moreover, the choice of ligand to the bioconjugation, such as an antibody or protein with a good affinity, allows the use of specific QDs for labeling DNA, proteins, or even cells (Chan and Nie 1998; Frasco and Chaniotakis 2010).

The majority of QDs that have been studied and used are those which in the core have selenium or tellurium atoms, because of the wide range of spectrum emitted by the region containing the atoms of these elements (Drbohlavova et al. 2009; Frasco and Chaniotakis 2010; Michalet et al. 2005; Smith et al. 2004). In order to reduce the cytotoxicity of QDs and improve their spectroscopic properties (e.g., elimination of photobleaching), different synthesis methods have been developed (Chan et al. 2002; Grieve et al. 2000; Michalet et al. 2005; Wang and Li 2007). Another way to reduce QDs cytotoxicity to an acceptable level is by coating their surface. The surface coating of QDs is mainly due to their stability, as without it they tend to aggregate and lose fluorescence. Additionally, it protects the nanocrystallites from chemical degradation. QDs may be safely used as fluorescent probes in biological labeling, markers for monitoring drug release, and for controlled modification of structural and functional properties of intracellular components (Alivisatos 1996; Chan et al. 2002; Chan and Nie 1998; Derfus et al. 2004; Dubois et al. 2007; Frasco and Chaniotakis 2010; Grieve et al. 2000; Hoshino et al. 2004; Michalet et al. 2005; Wang and Li 2007).

### Chip-CE technique

Capillary electrophoresis (CE) is often used to detect MT and to separate isoforms of MT (Minami et al. 2002; Richards and Beattie 1995; Ryvolova et al. 2012; Zalewska et al. 2011). CE techniques constitute a family of electrokinetic methods. During CE analysis through capillaries or micro and nanochannels, analytes migrate under the influence of the applied voltage. Separation in these methods is based on ionic mobility (Righetti et al. 2013; Sieradzka et al. 2014; Zhu et al. 2012). In addition, CE can also be used for monitoring MT concentration during illness, as well as analyzing stability and affinity in the conditions of an experimental environment (Petersen et al. 2003; Ryvolova et al. 2012; Swinney and Bornhop 2000). It is also proposed to apply this technique in experiments involving modification of MT and its ability to form aggregates and polymers (Krizkova et al. 2009a).

Due to the presence of two domains in the MT structure: β-domain (N-terminus, Me(II)_3_Cys_9_) and α-domain (C-terminus, Me(II)_4_Cys_11_) and the high affinity to bind Cd because of Cd-S bond formation, it is possible to observe the interaction of CdTe with MT (Skalickova et al. 2013; Thormann et al. 2001). For the determination of bioconjugates of QDs with MT, a technique is needed that allows for the simultaneous analysis of several samples with high efficiency in a relatively short time. Chip-CE can be used as a tool to effectively and efficiently analyze the marking of MT with QDs. To our knowledge, chip-CE has not been applied in the study of CdTe nanoparticles interaction with MT to date.

In this study, we have attempted to show the differences in the interaction of QDs with human MT, which depend on different sizes of QDs. The obtained complexes were analyzed using chip-CE and fluorescence intensity.

## Experimental

### Chemicals

Chemicals such as mercaptosuccinic acid (MSA), cadmium acetate, cadmium chloride (99.999 %), and sodium tellurite were purchased from Sigma Aldrich (St. Louis, MO, USA). Two stock solutions (50 μg/mL of Cd(II) and 500 μg/mL of MSA) were prepared daily and subsequently diluted to the appropriate concentration just prior to use. As a supporting electrolyte, 0.2 M acetate buffer of pH 5 was used. High-purity deionized water was used throughout the study (Milli-Q Millipore 18.2 MΩ/cm, Bedford, MA, USA).

### Sample preparation

Human liver metallothionein was obtained according to procedure described previously (Milnerowicz and Bizon 2010). Briefly, pieces of human liver were washed several times with PBS (the study protocol was approved by the Local Bioethics Committee (KB No 165/1999)). The tissue was homogenized in a buffer (10 mM Tris/HCl, pH 8.6, 10 mM β-mercaptoethanol and 25 mM sucrose) with a ratio of 1:4 (*v/v*). The resulting homogenate was centrifuged each time at 4 °C in three stages under the following conditions: 1—5000×*g* speed for 25 min, 2—speed of 10,000×*g* for 1 h, 3—speed 105,000×*g* for 2 h. The obtained supernatant was separated into two samples wherein one of them (Cd-MT) was saturated with CdCl_2_ and incubated in a water bath at 80 °C for 10 min and stirred. Both cytosol fractions (nonCd-MT and Cd-MT) were precipitated with acetone in scope from 60 % to 80 % (left overnight). After centrifugation, the resulting precipitates were dissolved in Tris-HCl pH 8.6 and centrifuged again. The samples were applied to a Sephadex G-75 column. Fractions containing MT were verified by SDS/PAGE, Western Blot, and ELISA (Milnerowicz and Bizon 2010). The samples containing human MT (both isoforms MT-1 and MT-2) were concentrated by Cut-off filter 3 K (Amicon Ultra-0.5 Centrifugal Filter Devices) prior to analysis on MALDI-TOF/TOF (supplementary material 2).

### CdTe QDs synthesis

**Fig. 1:**
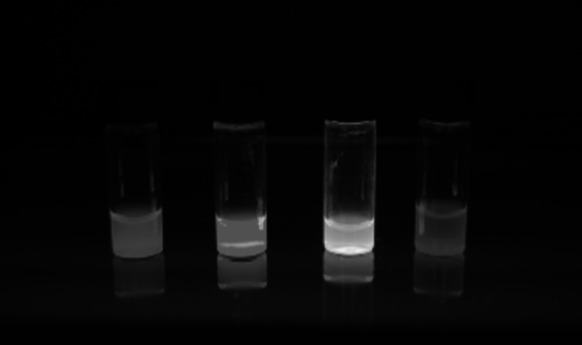
Picture shows synthetic *blue*, *green*, *yellow* and *red* QDs respectively after ilumination using UV transiluminator (254 nm). (Color figure online)

The method of CdTe QDs preparation was previously published by Duan et al. (2009). A spare solution of CdTe QDs was prepared by dissolving cadmium acetate dihydrate (0.044 g) in 76 mL of MiliQ water in a 200 mL beaker on a magnetic stirrer. Then, mercaptosuccinic acid (MSA) (60 mg) in water (1 mL) was added followed by 1.8 mL of 1 M NH_3_. Finally, a solution of Na_2_TeO_3_ (0.0055 g) in water was added and after a few minutes 40 mg of NaBH_4_ was poured into the stirred solution. The solution was stirred for 1 h, its volume was adjusted to 100 mL with the addition of water, after which it was heated in vials filled with 2 mL of the solution in a Multiwave 300 microwave oven (Anton Paar GmbH, Graz, Austria) (300 W, 10 min). CdTe QDs were prepared at various temperatures according to emission color (50 °C—blue, 70 °C—green; 90 °C—yellow; 130 °C—red, Fig. 1).

### Dynamic light scattering

The average particle hydrodynamic parameter and diameters distribution of the nanoparticles were determined by a Zetasizer (Malvern-zetasizer Nano ZS, Malvern, UK) at 25 °C. The measurement was performed after diluting the solution with deionized water. Size as hydrodynamic diameter is modeled by Stokes–Einstein equation (Murdock et al. 2008). The hydrodynamic parameter of quantum dots increased with the increasing temperature of the preparation process. The sizes (hydrodynamic diameters) of the nanoparticles were as follows: 3.4 nm (blue QDs), 3.8 nm (green QDs), 4.5 nm (yellow QDs), and 5.2 nm (red QDs).

### Conjugation of QDs with MT

4 μL of each sample of Cd-MT and nonCd-MT consisting of both isoforms (MT-1 and MT-2) at a concentration 0.1 mg/mL was mixed with 4 μL of each QDs. The mixture was incubated for 2 h at 20 °C in a thermoblock (Eppendorf thermomixer, Germany).

### Chip-CE

Analyses using an automated microfluidic Experion electrophoresis system (Bio-Rad, USA) were carried out according to the manufacturer’s instructions with supplied chemicals (Experion Pro260 analysis kit, Bio-Rad). The kit allows for fast and reproducible protein analysis with a separation range of 10–260 kD with 2.5 ng/µL sensitivity; detection was performed using colloidal Coomassie Blue gel staining. The presence of the ladder (as a standard) allowed correlation of migration time with molecular weight. A sample analysis was carried out according to the following steps:

1. Priming the chip—microchannels were filled in with the microfluidic gel-stain solution (with fluorescent dye). 0.2 μg of both MT samples (nonCd-MT and Cd-MT) was used per one chip-CE sample. A 4 μL of each sample was mixed with 2 μL of non-reducing sample buffer without heating (30 μL of the sample buffer with 1 μL of water), and 84 μL of water was added. The ladder was prepared in reducing conditions (30 μL of the sample buffer with 1 μL of β-mercaptoethanol (total concentration of β-mercaptoethanol is 3.3 % (v/v), 95 °C, 5 min). After priming the chip with the gel and gel-staining solution, the sample (6 μL) was loaded into the sample well. The Pro260 Ladder included in the kit was used as a standard.
2. The prepared chip was inserted into the electrophoresis station. During separation performed at 21 °C (voltage: 100–240 V), the fluorescent dye associated with the lithium dodecyl sulfate (LDS) coating the proteins.
3. As the molecules migrated towards the end of the separation channel, a laser excited the dye, causing it to fluorescence (Bousse et al. 2001).

### Fluorimetric measurement

**Fig. 2:**
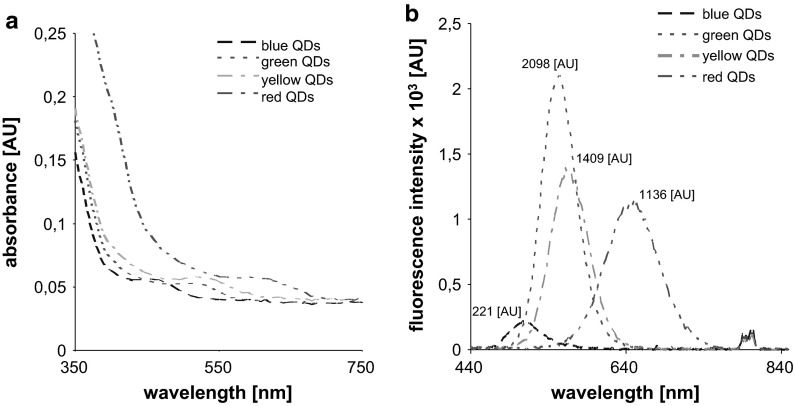
Absorbance (**a**) and fluorescence spectra (**b**) of *blue*, *green*, *yellow* and *red* QDs. (Color figure online)

Fluorescence and absorbance spectra were acquired with a multifunctional microplate reader EnSpire Multimode Reader (PerkinElmer, USA). The absorbance scan was measured within a range of 350–750 nm (Fig. 2a). Based on the results obtained, 400 nm was used as an excitation wavelength and the fluorescence scans were measured within the range from 440–840 nm per 5 nm steps (Fig. 2b). Each intensity value is an average of 5 measurements. 15 µL of each QDs were mixed with 15 µL of each sample of nonCd-MT and Cd-MT (consisting of two isoforms (MT-1 and MT-2) with the concentration of 0.1 μg). After 2 h of incubation, the mixed samples were placed in a transparent 96-well microplate with a flat bottom by Nunc (Thermo Scientific, USA).

## Results

**Fig. 3:**
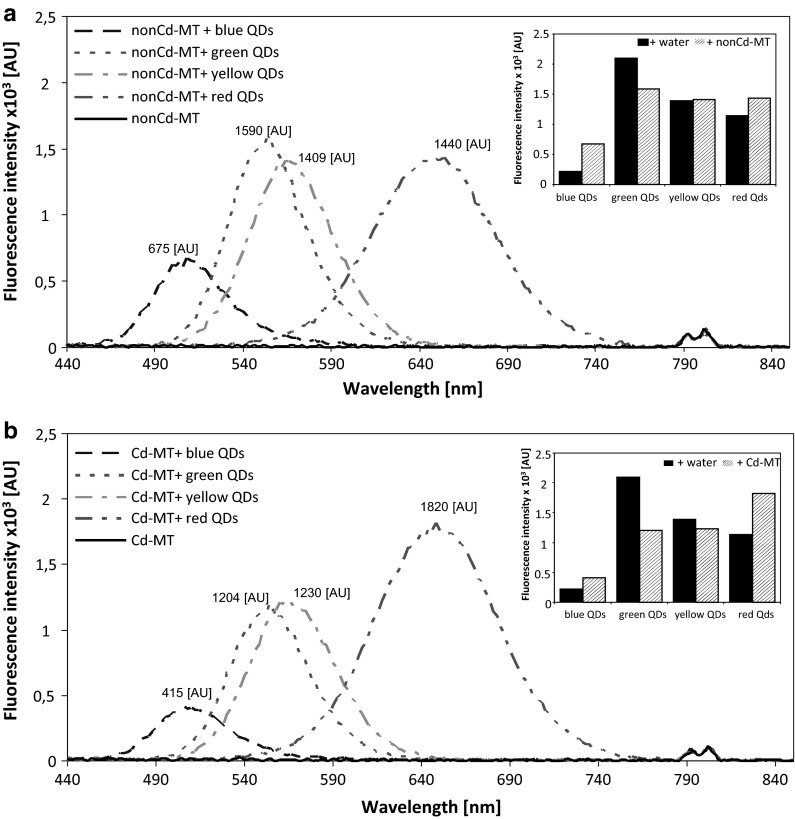
Fluorescence intensity of bioconjugated QDs with (**a**) nonCd-MT and (**b**) Cd-MT. In insets are shown the differences obtained in emission maxima. (Color figure online)

The synthesized QDs exhibited various colors after excitation with UV light (254 nm) (Fig. 1). The resulting products were characterized with the use of fluorescence spectroscopy (Fig. 2b). After interaction of QDs with Cd-MT and nonCd-MT (2 h incubation of each QD with each MT sample, for details see Material and Methods section), different fluorescent behaviors of Cd-MT and nonCd-MT was observed (Fig. 3). On the fluorescence intensity spectra, higher peaks were present for nonCd-MT with blue QDs (675 AU) and with red QDs (1440 AU) than for lone blue QDs (221 AU) and red QDs (1136 AU), respectively (Figs. 2b, 3a). The fluorescence intensity was the same for yellow QDs with nonCd-MT (1409 AU) and yellow QDs (1409 AU, Fig. 2b), whereas the peak obtained for green QDs with nonCd-MT (1590 AU) was lower than the peak for green QDs alone (2098 AU) (Fig. 3a).

The fluorescence intensity spectrum for Cd-MT shows higher peaks for Cd-MT with blue QDs and red QDs (415 AU and 1820 AU, respectively, Fig. 3b) compared to the peaks obtained for blue and red QDs alone (Fig. 2b). The peaks obtained for Cd-MT with green QDs (1204 AU) and yellow QDs (1230 AU) (Fig. 3b) were lower than the peaks obtained for green and yellow QDs alone (Fig. 2b).

On the fluorescence spectra, it was observed that the peak obtained for Cd-MT + red QDs (1820 AU) was higher than the peak obtained for nonCd-MT (1440 AU) (Fig. 3a, b). However, the peaks obtained for nonCd-MT with blue (675 AU), green (1590 AU), and yellow QDs (1409 AU) (Fig. 3a) were higher than the peaks obtained for Cd-MT with blue (415 AU), green (1204 AU), and yellow QDs (1230 AU), respectively (Fig. 2b).

**Fig. 4:**
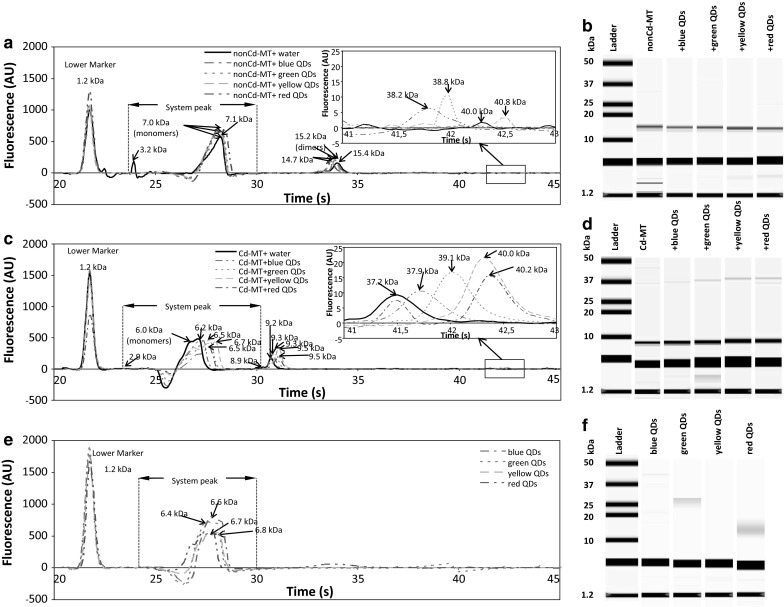
Electrophoreograms of: (**a**) bioconjugated nonCd-MT with QDs, (**c**) bioconjugated Cd-MT with QDs and (**e**) QDs. (**b**), (**d**) and (**f**) are a gel of nonCd-MT with QDs, Cd-MT with QDs and four QDs respectively. (Color figure online)

Interaction of Cd-MT and nonCd-MT with QDs was studied also using chip capillary electrophoresis. The chip-CE electropherogram for nonCd-MT shows three main peaks sized 3.2 kDa, 7.0 kDa, and 15.4 kDa corresponding to migration times at: 24.0, 28.2, and 34.0 s, respectively (Fig. 4a, b). The signal of the system peak for nonCd-MT characterized by a time shift in the migration time range of 23.7–30.0 s (1.9–7.0 kDa) could be compared to the ladder system peak, which was detected at 28 s (6.8 kDa) (supplementary material 2).

On the electropherograms with Cd-MT, three main peaks were observed with the sizes of 6.0, 9.2, and 40 kDa corresponding to migration times at 27.2, 30.7, and 41.5 s, respectively (Fig. 4c, d). Signal of the system peak for Cd-MT characterized by the time shift in migration time range 23.6–30.4 s (2.9–8.9 kDa) could be compared to the ladder system peak, which was detected at 28 s (6.8 kDa) (supplementary material 2).

The obtained peaks for both samples (nonCd-MT and Cd-MT) with Mr of roughly 6–7 kDa indicated the presence of MT monomers; however, the monomers co-migrated with a system peak, and thus, their correct sizing is not possible. For nonCd-MT, an additional peak with 15.4 kDa was noted, which may indicate the presence of dimers MT (Fig. 4a, b). For Cd-MT, an additional peak was noted at about 37–40 kDa which may indicate the presence of oligomers. The graphs show shifted peaks and differences in Mr for nonCd-MT monomers (Mr = 7.0 kDa, *t* = 30.0 s) and Cd-MT monomers (Mr = 6.0 kDa, *t* = 27.2 s). Furthermore, differences in the migration time (and Mr) for the second main peak were observed at 36.3 s (15.4 kDa) for nonCd-MT and at 30.7 s (9.2 kDa) for Cd-MT.

To prove that the signals observed for samples of MT bioconjugated with QDs were not derived from QDs, an electropherogram was performed for four different QDs (Fig. 4e, f). The peaks for blue, green, yellow, and red QDs alone corresponded to 6.6 kDa (*t* = 27.6 s), 6.4 kDa (*t* = 27.4 s), 6.8 kDa (*t* = 28.0 s), and 6.7 kDa (*t* = 27.6 s) Mr, respectively (Fig. 4e, f). The range migration times for systems peaks were observed at 24.5 s (2.5 kDa)–29.5 s (6.6 kDa) for blue QDs, 24.3 s (2.3 kDa)–29.7 s (6.7 kDa) for green QDs, 24.9 s (2.8 kDa)–29.8 s (6.9 kDa) for yellow QDs, and 24.8 s (2.7 kDa)–29.6 s (6.7 kDa) for red QDs (Fig. 4e). It was observed that the system peak obtained for nonCd-MT was shifted in time when compared to the system peaks obtained for each QDs (Fig. 4a, e).

The graphs in Fig. 4a show that the addition of CdTe QDs to nonCd-MT led to the intensities of the peaks at 28 s increasing, which may be explained by the formation of QDs-(nonCd-MT) complexes. The observed fluorescence intensity for peaks at the positions of roughly 28, 34, and 41 s for blue and red QDs-nonCd-MT complexes was higher than the fluorescence intensity obtained for others QDs+nonCd-MT complexes (Fig. 4a). It shows that nonCd-MT prefers to interact with blue and red QDs rather than with others QDs.

The peak at about 34 s (Mr = 15 kDa) may indicate the presence of MT dimers (Fig. 4a, b). The graphs show that MT dimers are likely to combine with green QDs rather than with blue ones (Fig. 4a), which corresponds to the larger increase in the peak obtained for green QDs-(nonCd-MT) complexes rather than blue QDs-(nonCd-MT) when compared to nonCd-MT.

In the case of Cd-MT, the addition of QDs (Fig. 4c, d) shows that peaks observed for Cd-MT complexes with QDs at 27 s decreased and shifted towards higher Mr in comparison with Cd-MT. It was observed that the largest number of Cd-MT particles bound to the smallest sized QDs (blue QDs) (Fig. 4c). Intensities of the peaks for complexes of Cd-MT with green and yellow QDs observed at 31 s were slightly increased, while intensities for the complexes of Cd-MT with blue and red QDs were comparable to Cd-MT alone, but with time shifted towards higher Mr depending on their size (Fig. 4c). For green QDs, the obtained peak occurred later in time, which means that larger aggregates of cadmium MT are more likely to combine with them than with the smaller ones (blue CdTe) or the largest ones (red CdTe, Fig. 4c, d).

At the position of roughly 41 s (Mr 37.2 kDa for Cd-MT) (Fig. 4c), new peaks occurred for all complexes of QDs with Cd-MT (Fig. 3c, d). Increased intensities at 41–43 s were observed for complexes of Cd-MT with yellow (the highest) and green and red (similar intensities) QDs compared with Cd-MT alone (*t* = 41.5 s, 37.2 kDa). At 42–43 s, a time shift towards higher Mr for all QDs was also observed (e.g., 39.1 kDa for Cd-MT+green QDs, 40.0 kDa for Cd-MT+yellow QDs) (Fig. 4c).

## Discussion

A large number of publications have been reported on the validity of the determination of MT as a tumor marker (Cherian et al. 2003; Krizkova et al. 2010b; Krizkova et al. 2011). An important factor would be the selection of such a method that would allow for both sensitive and selective analysis of MT presence in body fluids.

The application of QDs as a marker for peptides (Rosenthal et al. 2011), proteins (Miyawaki et al. 2003) or DNA (Mitchell et al. 1999) is well known. Using luminescence QDs as a tumor biomarker seems to be an effective method of tagging at the molecular level (Chan et al. 2002; Gao et al. 2005; Mitchell et al. 1999). The mechanisms by which QDs bind to biomolecules are still being explored (Gao et al. 2005; Mitchell et al. 1999; Miyawaki et al. 2003; Pons et al. 2006). It is worth mentioning the fact that the type of interaction depends on the geometric orientation of biomolecules and hydrodynamic sizes of QDs (Michalet et al. 2005; Pons et al. 2006). Researchers point out that QDs can interact with proteins through the presence of cysteine residues (Miyawaki et al. 2003; Rosenthal et al. 2011). The use of QDs in medicine and biochemistry appears to be a promising technique that allows for quick labeling and analysis of proteins. The advantage of this technique seems to be the ability to control and influence the properties of QDs through manipulation in the selection of ligand or a change of reaction (Chan et al. 2002; Michalet et al. 2005). In addition, QDs can provide usable imagery due to their fluorescent properties. It is important to ensure the safety and reduce the cytotoxicity of these probes, for example, by decreasing the surface defect density (Silva et al. 2014). In the cited article, the authors showed that increasing concentrations of Se reduced the toxic effects of CdSe QDs. It was noted that due to the affinity of cadmium to MT, the interaction of this protein with CdTe QDs is possible, and that reduced presence of Cd^2+^ diminished the expression of MT mRNA (Silva et al. 2014).

The interaction of MT with QDs is known, and it has been described using spectrophotometry, fluorimetry and voltammetry (Skalickova et al. 2013). Through spectral and electrochemical techniques, it was possible to locate signals of MT after interaction with QDs in Mr corresponding to MT dimers or trimers (Tmejova et al. 2014). Oligomerization of MT after interaction with metals or after oxidation was previously studied with CE, MS (Hong et al. 2000; Shen et al. 2006), spectrophotometry (Palumaa and Vasak 1992), and chip-CE (Krizkova et al. 2010a). The interaction of MT with QDs can occur not only under in vitro conditions but also in living organisms, as a part of heavy metals detoxification resulting in biosynthesis of MT-stabilized QDs (Skalickova et al. 2013; Sturzenbaum et al. 2013; Trabelsi et al. 2013).

The chip-CE technique was proposed for the first time for analysis of MT by Krizkova et al. (Krizkova et al. 2009a). The authors showed that this technique can be used to analyze MT, including to investigate the effects of oxidation on the structure of MT. Many commercial platforms for chip electrophoresis allowing routine analysis of nucleic acids or proteins are available, and their use for NPs analysis has been tested (Smejkal and Foret 2012). One such example is the use of this technique for the detection of AgNPs in common water (Chua and Pumera 2013).

The interactions of MT with QDs described above were analyzed using various techniques, and the chip-CE technique was used to analyze MT as well as QDs, but to our knowledge, chip-CE has not previously been used for studying interactions of MT with CdTe QDs. Our results indicate that this technique is suitable for monitoring proteins interacting with NPs. The observed increased intensity of peaks for MT-QDs complexes allows for the use of chip-CE to detect QDs binding to MT. Unfortunately, due to the presence of system peaks, an accurate analysis of the MT monomer is not possible, and it is suspected that a peak derived from the MT monomer is blocked by the system peaks (Krizkova et al. 2009a). Nevertheless, the signal corresponding to the amount of the protein bound or unbound with QDs allows determination of binding of MT-QDs depending on the size of CdTe. The chip-CE technique successfully enabled the imaging bioconjugation of these QDs with MT, which can be used in monitoring and identification of this protein. In this study, we used chip-CE to verify complexes generated in the process of bioconjugation of different sizes of CdTe with MT. The interaction between MT and QDs was also verified using fluorescence intensity.

As shown in this report, MT readily combines with the proposed CdTe due to its affinity for Cd. While in nonCd-MT complexes with QDs, only slight changes in peaks’ intensities were observed, in Cd-MT, it was observed that additional changes in peaks’ time migration appeared, resulting in a shift to higher Mr for Cd-MT complexes with QDs (depending on the hydrodynamic diameter of the QDs) compared to MT alone. The changes in migration time were most distinctive for green and yellow QDs. Moreover, for Cd-MT, we observed new peaks in positions of 37 kDa, which were shifted depending on the size of QDs. The choice of the size of CdTe QDs allowed us to analyze not only the MT monomers but also the MT oligomers in the sample.

The increase of 31 s peak at Cd-MT-QDs indicated that MT dimers (10 and 15 kDa) exhibit a higher affinity with QDs in a Cd-saturated state and form oligomers (40 kDa) depending on QDs size (for Cd-MT+red QDs complexes, the intensity decreased at 27 and 31 s but at 41 s increased compared to Cd-MT alone). Also, on the fluorescence intensity spectra, it can be noticed that the peak obtained for Cd-MT+red QDs was higher than the peak obtained for nonCd-MT+red QDs. This may be connected with Cd-MT saturation with heavy metals, and thus structure stabilization of MT and a preferable formation of aggregates (observed on chip-CE electropherogram at 41–43 s). The peaks of fluorescence intensity obtained for blue, green, and yellow QDs conjugated with nonCd-MT were higher than the peaks obtained for the same QDs with Cd-MT. This can be explained by greater affinity for nonCd-MT to bind Cd from QDs than Cd-MT (due to the saturated binding sites of this metal) (Carpene et al. 2007; Krizkova et al. 2010a).

Based on these results and taking into account the role of MT as a prognostic marker in cancerogenesis, chip-CE can be used as a technique to verify interaction between protein and NPs.

## Electronic supplementary material

Supplementary material 1 (ppt 149 kb)

Supplementary material 2 (ppt 421 kb)

## Abbreviations

CE: Capillary electrophoresis
LDS: Lithium dodecyl sulfate
Mr: Molecular weight
MT: Metallothionein
NPs: Nanoparticles
QDs: Quantum dots
