# Supplementary figures and images for: Fluorescence-tagged metallothionein with CdTe quantum dots analyzed by the chip-CE technique
Source: J Nanopart Res. 2015 Oct 28;17(11):423. doi: 10.1007/s11051-015-3226-8 (PMC4624813; doi:10.1007/s11051-015-3226-8)

## Slide 1
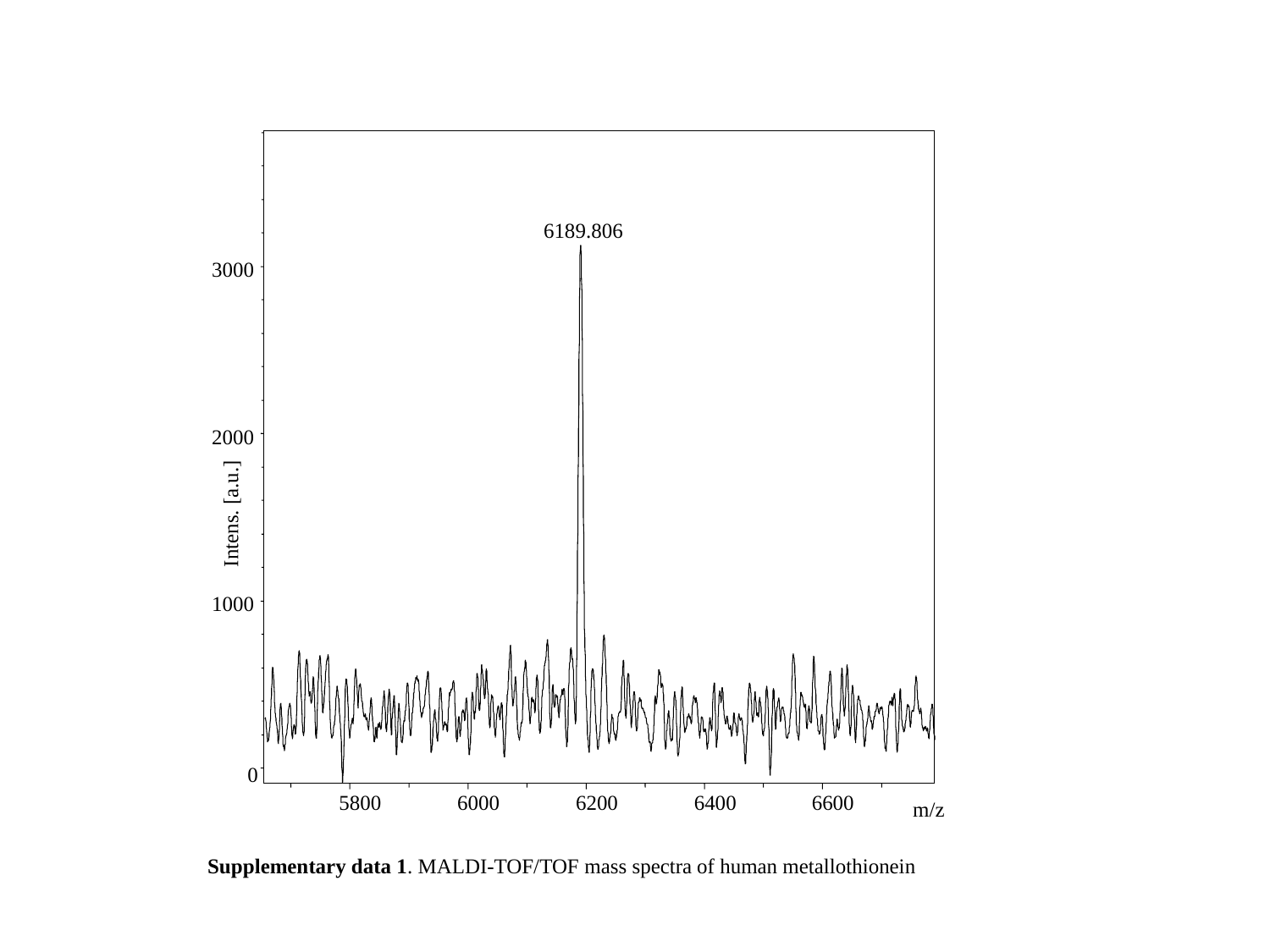

6189.806
3000
2000
Intens. [a.u.]
1000
0
5800
6000
6200
6400
6600
m/z
Supplementary data 1. MALDI-TOF/TOF mass spectra of human metallothionein

Supplement: Supplementary file 1 — Supplementary material 1 (ppt 149 kb) [file 11051_2015_3226_MOESM1_ESM.ppt]
