# Supplementary material for: Fluorescence-tagged metallothionein with CdTe quantum dots analyzed by the chip-CE technique
Source: J Nanopart Res. 2015 Oct 28;17(11):423. doi: 10.1007/s11051-015-3226-8 (PMC4624813; doi:10.1007/s11051-015-3226-8)
Supplement: Supplementary file 2 — Supplementary material 2 (ppt 421 kb) [file 11051_2015_3226_MOESM2_ESM.ppt]

## Slide 1
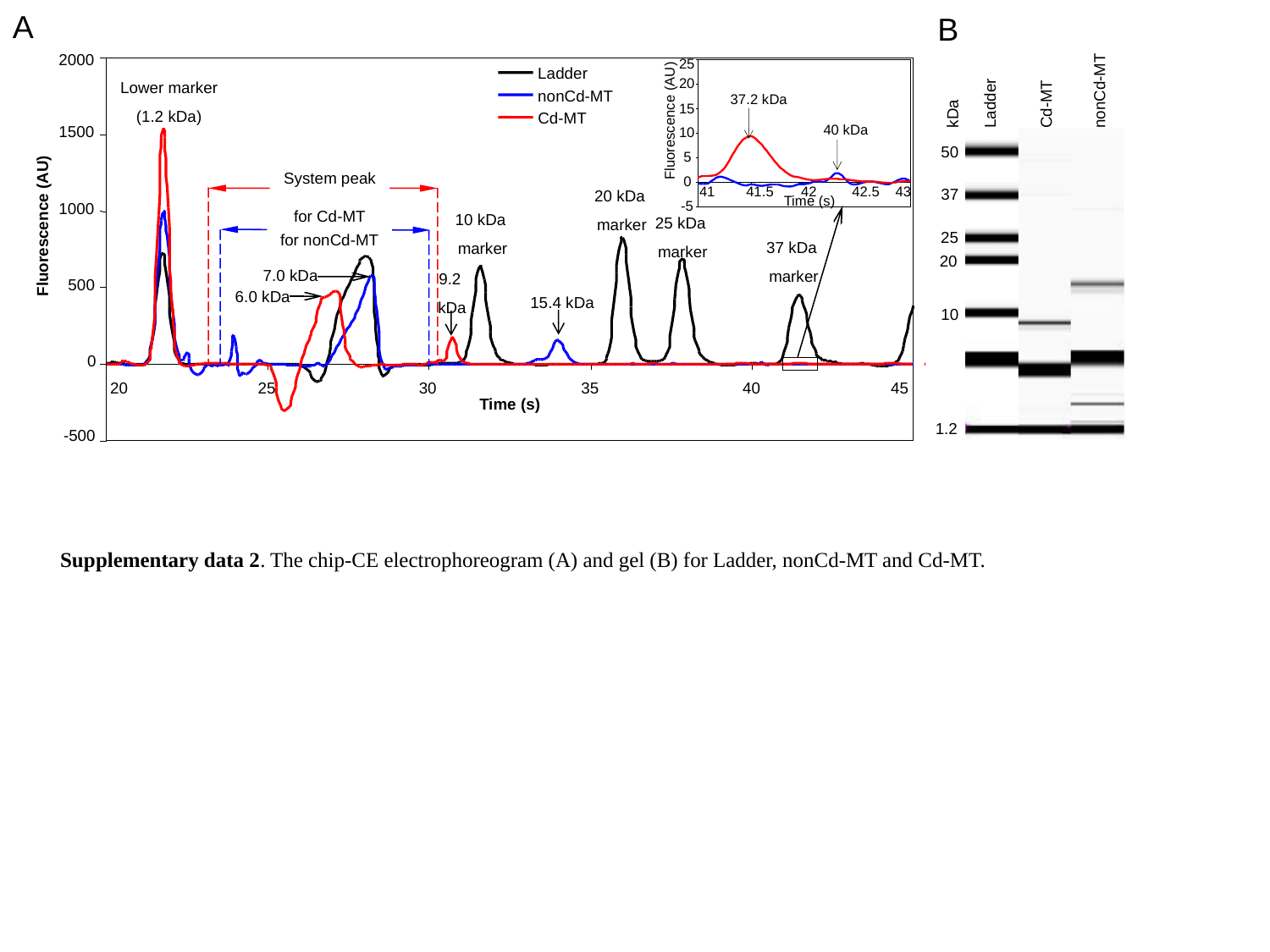

A
B
2000
25
Ladder
Lower marker
(1.2 kDa)
Cd-MT
nonCd-MT
20
Ladder
37.2 kDa
nonCd-MT
kDa
15
Cd-MT
Fluorescence (AU)
40 kDa
1500
10
50
5
System peak
for Cd-MT
0
37
20 kDa
marker
41
41.5
42
42.5
43
Time (s)
-5
1000
10 kDa
marker
System peak
for nonCd-MT
25 kDa
marker
Fluorescence (AU)
25
37 kDa
marker
20
7.0 kDa
9.2
kDa
500
6.0 kDa
15.4 kDa
10
0
20
25
30
35
40
45
Time (s)
1.2
-500
Supplementary data 2. The chip-CE electrophoreogram (A) and gel (B) for Ladder, nonCd-MT and Cd-MT.
